# Intermediate conduction velocity in two cases of Charcot−Marie−Tooth disease type 1A

**DOI:** 10.1111/ene.16199

**Published:** 2024-02-26

**Authors:** Pedro José Tomaselli, Julian Blake, James M. Polke, Osvaldo José Moreira do Nascimento, Mary M. Reilly, Wilson Marques Júnior, Matilde Laurá

**Affiliations:** ^1^ Clinical Hospital of Ribeirão Preto, Department of Neurosciences and Behaviour Sciences University of São Paulo Ribeirão Preto Brazil; ^2^ Centre for Neuromuscular Diseases, UCL Queen Square Institute of Neurology University College London London UK; ^3^ Department of Clinical Neurophysiology Norfolk and Norwich University Hospital Norwich UK; ^4^ UCLH Neurogenetics Laboratory National Hospital for Neurology and Neurosurgery London UK; ^5^ Neurology Department of Fluminense Federal University (UFF) Rio de Janeiro Brazil

**Keywords:** Charcot−Marie−Tooth disease, electromyography, hereditary sensory and motor neuropathy, *PMP22*

## Abstract

**Background and purpose:**

Charcot−Marie−Tooth disease type 1A (CMT1A) is the most prevalent hereditary neuropathy worldwide and classically has slow nerve conduction velocity (NCV), in most cases below 38 m/s. Two unrelated patients with motor NCVs in the upper limbs above 38 m/s are reported.

**Method:**

Case report.

**Results:**

Two genetically confirmed CMT1A patients are presented, from two unrelated families (one of British origin and the other of Brazilian origin). Both individuals had upper limb motor NCVs above 38 m/s, with values ranging from 41.9 to 45 m/s in the median nerve and from 42 to 42.3 m/s in the ulnar nerve. They presented with a very mild phenotype, with CMT Neuropathy Score version 2 (CMTNSv2) of 6 and 5, respectively. In contrast, affected family members within both kinships exhibited a classical phenotype with more severe disease manifestation (CMTNSv2 ranging from 12 to 20) and motor NCVs below 30 m/s.

**Conclusion:**

These cases, although very rare, highlight the importance of testing *PMP22* duplication in patients with intermediate conduction velocities.

## BACKGROUND

Charcot−Marie−Tooth disease type 1A (CMT1A) due to *PMP22* duplication is the most prevalent hereditary neuropathy worldwide [1]. Overexpression of *PMP22* leads to abnormal myelination of the peripheral nerves in humans and animal models, resulting in similar electrophysiological and neuropathological characteristics. One of the distinctive features of CMT1A is the uniform slowing of nerve conduction velocities (NCVs), reflecting the underlying processes of demyelination and remyelination. NCV slowing is fully penetrant and stable after the age of 2 [2]. Typically, median and ulnar motor NCVs (MNCVs) range between 10 and 38 m/s, with the majority showing values between 15 and 30 m/s. Only very few CMT1A patients have been reported having NCVs above 38 m/s [3, 4]. Consequently, diagnostic algorithms for hereditary peripheral neuropathy recommend genetic testing for *PMP22* duplication primarily in patients with upper limb MNCVs below 38 m/s.

## CASES

The probands from two unrelated families with genetically confirmed CMT1A due to *PMP22* duplication and upper limb MNCVs greater than 40 m/s were identified from the National Hospital for Neurology and Neurosurgery, Queen Square, London (family 1 III.1), and the Clinical Hospital of Ribeirão Preto, University of São Paulo (family 2 III.2). They were both tested by multiplex ligation‐dependent probe amplification which confirmed the presence of the classical 1.5 Mb duplication in chromosome 17. Additional *PMP22* Sanger sequencing was normal in both patients. Clinical and electrophysiological features are summarized in Table 1. Patient III.1 from family 1 reported clumsiness in her first decade of life. Later in life she experienced difficulties with hand dexterity and altered sensation in the right big toe. Her symptoms were very slowly progressive. At the age of 26 examination demonstrated very mild distal weakness in the upper and lower limbs and reduced pinprick sensation in the toes of the right foot. Neurophysiology of the median nerve showed prolonged distal motor latencies and slow MNCV (45 m/s). Ulnar MNCV was similarly slow (42 m/s). CMT Neuropathy Score version 2 (CMTNSv2) was 6 indicating mild clinical impairment. In her family her mother and maternal aunt were more severely affected (II.1 and II.2) and had typical MNCVs (22–32 m/s).

**TABLE 1:** Clinical features and neurophysiology.

	Family 1			Family 2			
	III.1 (proband)	II.1	II.2	III.2 (proband)	III.4	II.2	IV.2
Age of onset	First decade	First decade	First decade	20s	Second decade	Asymptomatic	7
Age at examination	26	61	51	26	32	50	8
Presenting symptoms	Last in races	Not good at sport	High arches/frequent falls	Difficulty walking, LL burning pain	Difficulty walking	Age 40 cerebellar stroke. After stroke frequent falls	Difficulty walking
UL muscle weakness (R, L)	FDIO 4+, 4+ APB 4+, 4+	FDIO 3, 3 APB 2, 3	FDIO 5, 4+ APB 4, 4+	No	FDIO 5, 4+ APB 4‐, 4‐	FDIO 4, 4 APB 4, 4	FDIO 4, 4
LL muscle weakness (R, L)	ADF 4+, 4+	ADF 4‐, 3	ADF 4+, 4	ADF 4+	ADF 4+, 4	ADF 4+, 4+	ADF 4, 4
UL deep tendon reflexes	Present	Present	Present	Present	Present with reinforcement	Present	Absent
LL deep tendon reflexes	Present	Absent	Knee present Ankle absent	Present	Present with reinforcement	Absent	Absent
Pinprick sensation	Normal	Ankles	Ankles	Normal	Wrists/ ankles	Mid forearm/knees	Normal/above ankles
Vibration sense	Normal	Knees	Ankles	Normal	Ankles	Knees	Normal
Joint position sense	Normal	Normal	Normal	Normal	Normal	Normal	Normal
CMTESv2	6	14	8	4	9	10	5
CMTNS v2	6	20	14	5	12	13	6
Motor nerve conduction studies							
Median DML ms, RR ≤ 4.0	5.7	10.1	9.7	7.65	6.4	7.65	8.3
Median amplitude mV, RR > 4.5	6.8	0.8	5.8	5.8	7.24	5.35	6.5
Median CV m/s, RR ≥ 50	45	22	30	41.9	25.9	24.4	24.7
Ulnar DML ms, RR < 3.9	4.1	7.5	6.9	3.95	4.6	4.6	NR
Ulnar amplitude mV, RR ≥ 6.0	7.8	0.4	6.5	5.03	7.9	4.8	NR
Ulnar CV m/s, RR ≥ 50	42	27	30	42.3	21.8	21.5	NR
Peroneal DML ms, NR < 5.9	6.3	Absent	12	6.25	5.5	5.45	NR
Peroneal amplitude mV, NR > 2.5	5.4	Absent	1.6	8.26	3.49	2.98	NR
Peroneal CV m/s, RR ≥ 40.0	25	Absent	24	34.2	18.1	19.0	NR
Sensory nerve conduction studies							
Median amplitude μV, RR ≥ 9	9	Absent	Absent	NR	5.0	3.2	NR
Median CV m/s, RR ≥ 50	41	Absent	Absent	NR	25.2	21.1	NR
Ulnar amplitude μV, RR ≥ 8	3	Absent	Absent	3.1	3.0	4.0	NR
Ulnar CV m/s, RR ≥ 50	43	Absent	Absent	35.7	20.4	25.5	NR
Radial amplitude μV, RR ≥ 15	33	Absent	Absent	11.1	4.1	5.8	11
Radial CV m/s, RR ≥ 50	55	Absent	Absent	52.2	19.6	25.6	31
Sural amplitude μV, RR ≥ 5	6	Absent	Absent	12.3	NR	3.0	NR
Sural CV m/s, RR ≥ 40	30	Absent	Absent	28.1	NR	27.5	NR

Abbreviations: ADF, ankle dorsiflexion; APB, abductor pollicis brevis; CMTES/CMTNSv2, Charcot−Marie−Tooth Examination/Neuropathy Score version 2; CV, conduction velocity; DML, distal motor latency; FDIO, first dorsal interosseous; LL, lower limb; NR, not recorded; RR, reference range; UL, upper limb.

The proband (III.2) from family 2 had an active and normal lifestyle during the first two decades of life. From the age of 24 he developed progressive distal burning pain and difficulty walking. Neurological examination at the age of 26 showed a normal gait, very mild distal weakness in the right lower limb and normal sensory examination. Median MNCV was 41.9 m/s and ulnar MNCV was 42.3 m/s. Radial sensory conduction velocity was normal (52.2 m/s) with reduced amplitude (11.1 μV). Sural amplitude was normal (12.3 μV) but with slow conduction velocity (28.1 m/s). An acquired neuropathy was suspected due to non‐uniform sensory velocity slowing but blood tests and cerebrospinal fluid analysis were unremarkable and the symptoms did not respond to treatment with high dose of intravenous methylprednisolone for 6 months. Subsequently his 8‐year‐old son (2.IV.2) presented with a slowly progressive length‐dependent demyelinating sensory‐motor neuropathy due to *PMP22* duplication. Other affected family members from family 2 (III.3, II.2) also had typical CMT1A phenotype.

## DISCUSSION

Two unrelated patients with genetically confirmed CMT1A and upper limb MNCVs greater than 40 m/s are described. Upper limb MNCVs of 38 m/s is the accepted cut‐off value between demyelinating (CMT1) and axonal CMT (CMT2). CMT1A demyelinating neuropathy typically presents with uniform conduction slowing and most patients have conduction velocities below 35 m/s [2].

Motor NCVs greater than 35 m/s were considered unlikely to be associated with *PMP22* duplication in a large cohort of 787 CMT patients [5]. All studies [1, 3, 4, 5], except two, reported velocities below 38 m/s in CMT1A patients. Kaku et al. [3] reported an average of median MNCVs of 23.9 m/s ranging from 10 to 42 m/s, with two patients having MNCVs greater than 38 m/s, including an adolescent and another individual in his early 70s, and Krampitz et al. [4] reported a case of a 45‐year‐old woman with CMT1A and median MNCV 42.8 m/s.

Two further cases with a very mild CMT1A phenotype and intermediate MNCVs (41.9–45 m/s) are reported. Interestingly, their affected family members had the classical clinical and electrophysiological CMT1A phenotype. It is hypothesized that the mechanism underlying this variability might be related to genetic modifying factors [6]. However, no further genetic analysis was performed in our two patients except sequencing of the *PMP22* gene which was normal. These cases confirm previous reports of clinical variability of the disease even within the same family and suggest that CMT patients with intermediate upper limb motor conduction velocities including values above 38 m/s should be screened for a *PMP22* duplication.

## CONFLICT OF INTEREST STATEMENT

The authors have no conflicts of interest to declare.

## Data Availability

The data that support the findings of this study are available on request from the corresponding author. The data are not publicly available due to privacy or ethical restrictions.

## References

[1] Wise CA , Garcia CA , Davis SN , et al. Molecular analyses of unrelated Charcot−Marie−Tooth (CMT) disease patients suggest a high frequency of the CMT1A duplication. Am J Hum Genet. 1993;53:853‐863. 8105684 PMC1682385

[2] García A , Combarros O , Calleja J , Berciano J . Charcot−Marie−Tooth disease type 1A with 17p duplication in infancy and early childhood: a longitudinal clinical and electrophysiologic study. Neurology. 1998;50(4):1061‐1067. 9566395 10.1212/wnl.50.4.1061

[3] Kaku DA , Parry GJ , Malamut R , Lupski JR , Garcia CA . Nerve conduction studies in Charcot−Marie−Tooth polyneuropathy associated with a segmental duplication of chromosome 17. Neurology. 1993;43(9):1806‐1808. 8414036 10.1212/wnl.43.9.1806

[4] Krampitz DE , Wolfe GI , Fleckenstein JL , Barohn RJ . Charcot−Marie−Tooth disease type 1A presenting as calf hypertrophy and muscle cramps. Neurology. 1998;51:1508‐1509. 9818900 10.1212/wnl.51.5.1508

[5] Saporta ASD , Sottile SL , Miller LJ , Feely SME , Siskind CE , Shy ME . Charcot−Marie−Tooth (CMT) subtypes and genetic testing strategies. Ann Neurol. 2011;69(1):22‐33. 21280073 10.1002/ana.22166 PMC3058597

[6] Tao F , Beecham GW , Rebelo AP , et al. Variation in SIPA1L2 is correlated with phenotype modification in Charcot−Marie−Tooth disease type 1A. Ann Neurol. 2019;85(3):316‐330. doi:10.1002/ana.25426 30706531 PMC7263419

